# Supplementary material for: Disruption in structural–functional network repertoire and time-resolved subcortical fronto-temporoparietal connectivity in disorders of consciousness
Source: eLife. 2022 Aug 2;11:e77462. doi: 10.7554/eLife.77462 (PMC9385205; doi:10.7554/eLife.77462)
Supplement: Supplementary file 1. — (A) Details about the patient population. (B) Brain regions involved in the extracted networks by non-negative tensor factorisation. [file elife-77462-supp1.docx]

**Supplementary File 1**

**Supplementary Table 1A.** MCS and UWS patients’ demographic and clinical characteristics. The table includes condition, etiology (traumatic brain injury (TBI) grouped with Haemorrhage Stroke and other focal leasons and Anoxia), time science injury (TSI), age, gender (F=female, M=male), Coma Recovery Scale-Revised (CSR-R) auditory, visual, motor, verbal, communication and arousal subscores, CRS-R total score.

| **Patient ID** | **Etiology** | **TSI (Days)** | **Age (Years)** | **Gender** | **Auditory** | **Visual** | **Motor** | **Verbal** | **Communication** | **Arousal** | **Total CSR-R** |
| --- | --- | --- | --- | --- | --- | --- | --- | --- | --- | --- | --- |
| MCS P1 | TBI | 3034 | 34 | F | 3 | 3 | 2 | 2 | 0 | 2 | 12 |
| MCS P2 | TBI | 1294 | 40 | F | 2 | 3 | 2 | 2 | 0 | 2 | 11 |
| MCS P3 | Anoxia | 13 | 62 | M | 0 | 3 | 2 | 1 | 0 | 1 | 7 |
| MCS P4 | TBI | 389 | 59 | F | 2 | 1 | 2 | 1 | 0 | 2 | 8 |
| MCS P5 | TBI | 589 | 30 | M | 3 | 2 | 2 | 2 | 0 | 1 | 10 |
| MCS P6 | TBI | 521 | 28 | M | 1 | 3 | 2 | 2 | 0 | 2 | 10 |
| MCS P7 | TBI | 533 | 47 | M | 3 | 5 | 2 | 1 | 0 | 2 | 13 |
| MCS P8 | Anoxia | 2639 | 38 | M | 1 | 3 | 2 | 2 | 0 | 1 | 9 |
| MCS P9 | TBI | 2690 | 24 | M | 3 | 3 | 5 | 1 | 0 | 2 | 14 |
| MCS P10 | Anoxia | 9900 | 39 | M | 3 | 3 | 5 | 2 | 0 | 2 | 15 |
| MCS P11 | Anoxia | 314 | 26 | F | 3 | 1 | 2 | 1 | 0 | 2 | 9 |
| MCS P12 | TBI | 407 | 31 | M | 0 | 1 | 5 | 2 | 0 | 1 | 9 |
| MCS P13 | Anoxia | 64 | 29 | M | 1 | 3 | 2 | 2 | 0 | 2 | 10 |
| MCS P14 | Anoxia | 639 | 43 | M | 2 | 3 | 1 | 2 | 0 | 2 | 10 |
| MCS P15 | TBI | 1241 | 53 | M | 0 | 3 | 2 | 2 | 0 | 2 | 9 |
| MCS P16 | TBI | 135 | 51 | M | 3 | 4 | 2 | 1 | 0 | 2 | 12 |
| MCS P17 | TBI | 1383 | 68 | F | 3 | 1 | 3 | 2 | 0 | 2 | 11 |
| MCS P18 | TBI | 1331 | 35 | M | 3 | 0 | 2 | 1 | 0 | 2 | 8 |
| MCS P19 | TBI | 35 | 73 | M | 0 | 2 | 0 | 1 | 0 | 1 | 4 |
| MCS P20 | Anoxia | 104 | 43 | F | 3 | 1 | 3 | 1 | 0 | 0 | 8 |
| MCS P21 | Anoxia | 401 | 29 | M | 1 | 3 | 2 | 1 | 0 | 2 | 9 |
| MCS P22 | TBI | 255 | 39 | M | 4 | 5 | 4 | 2 | 1 | 2 | 18 |
| MCS P23 | Anoxia | 1482 | 32 | M | 3 | 4 | 2 | 2 | 1 | 2 | 14 |
| MCS P24 | TBI | 641 | 23 | M | 3 | 3 | 0 | 1 | 0 | 2 | 9 |
| MCS P25 | Anoxia | 34 | 47 | F | 3 | 4 | 5 | 3 | 2 | 2 | 19 |
| MCS P26 | Anoxia | 7814 | 34 | M | 1 | 3 | 5 | 2 | 0 | 2 | 13 |
| MCS P27 | TBI | 20 | 52 | M | 3 | 3 | 2 | 2 | 1 | 2 | 13 |
| MCS P28 | TBI | 37 | 26 | F | 2 | 3 | 3 | 0 | 1 | 1 | 10 |
| MCS P29 | TBI | 242 | 46 | F | 2 | 3 | 2 | 1 | 0 | 2 | 10 |
| MCS P30 | Anoxia | 396 | 57 | M | 3 | 0 | 2 | 2 | 0 | 2 | 9 |
| UWS P1 | TBI | 283 | 52 | F | 1 | 0 | 2 | 2 | 0 | 1 | 6 |
| UWS P2 | Anoxia | 743 | 30 | M | 1 | 0 | 2 | 1 | 0 | 2 | 6 |
| UWS P3 | Anoxia | 92 | 74 | M | 1 | 0 | 1 | 1 | 0 | 1 | 4 |
| UWS P4 | TBI | 43 | 64 | M | 1 | 0 | 2 | 1 | 0 | 1 | 5 |
| UWS P5 | Anoxia | 18 | 20 | M | 1 | 0 | 0 | 1 | 0 | 1 | 3 |
| UWS P6 | Anoxia | 304 | 60 | F | 1 | 1 | 1 | 1 | 0 | 2 | 6 |
| UWS P7 | Anoxia | 29 | 44 | M | 1 | 0 | 1 | 0 | 0 | 2 | 4 |
| UWS P8 | Anoxia | 38 | 50 | F | 0 | 0 | 0 | 2 | 0 | 1 | 3 |
| UWS P9 | Anoxia | 50 | 69 | F | 0 | 1 | 2 | 1 | 0 | 1 | 5 |
| UWS P10 | Anoxia | 1868 | 21 | F | 1 | 0 | 1 | 2 | 0 | 2 | 6 |
| UWS P11 | Anoxia | 129 | 49 | F | 1 | 0 | 0 | 1 | 0 | 2 | 4 |
| UWS P12 | TBI | 24 | 58 | M | 0 | 1 | 2 | 0 | 0 | 1 | 4 |
| UWS P13 | Anoxia | 30 | 44 | M | 1 | 1 | 1 | 1 | 0 | 1 | 5 |
| UWS P14 | Anoxia | 335 | 40 | F | 1 | 0 | 2 | 1 | 0 | 2 | 6 |

**Supplementary Table 1B.** Brain Region Involved for every networks illustrated in the figure I to N extracted from NNFT based on Shen et al. (2015) atlas labeling.

| **Brain Region Involved for DMN** | **Intensity** |
| --- | --- |
| 44% Frontal_Med_VMPFC_R [26] / 31% Cingulum_Ant_R [32] | 0.90924 |
| 60% Frontal_Sup_Medial_R [24] / 20% Cingulum_Ant_R [32] | 0.93139 |
| 52% Frontal_Sup_R [4] / 41% Frontal_Sup_Medial_R [24] | 0.80972 |
| 87% Angular_R [66] | 0.84221 |
| 44% Cingulum_Post_R [36] / 39% Cingulum_Mid_R [34] | 0.79147 |
| 57% Precuneus_R [68] / 32% Calcarine_R [44] | 0.70205 |
| 79% Precuneus_R [68] | 0.88825 |
| 54% Frontal_Med_VMPFC_L [25] / 31% Cingulum_Ant_L [31] | 0.91556 |
| 50% Frontal_Sup_Medial_L [23] / 47% Cingulum_Ant_L [31] | 0.96556 |
| 40% Frontal_Sup_Medial_L [23] / 30% Frontal_Sup_L [3] | 0.74546 |
| 57% Frontal_Sup_Medial_L [23] / 41% Frontal_Sup_L [3] | 0.85587 |
| 44% Frontal_Sup_Medial_L [23] / 43% Frontal_Sup_L [3] | 0.83064 |
| 59% Angular_L [65] / 26% Parietal_Inf_L [61] | 0.76956 |
| 55% Temporal_Mid_L [85] / 27% Angular_L [65] | 0.77156 |
| 77% Temporal_Mid_L [85] | 0.80842 |
| 45% Precuneus_L [67] / 34% Calcarine_L [43] | 0.82173 |
| 74% Precuneus_L [67] | 1 |
| **Brain Region Involved for Visual** | **Intensity** |
| 39% Fusiform_R [56] / 25% Occipital_Inf_R [54] | 0.70872 |
| 51% Lingual_R [48] / 33% Fusiform_R [56] | 0.87274 |
| 67% Occipital_Mid_R [52] | 0.88126 |
| 42% Occipital_Sup_R [50] / 26% Cuneus_R [46] | 0.79673 |
| 48% Lingual_R [48] / 17% Fusiform_R [56] | 0.83135 |
| 65% Cuneus_R [46] | 0.84935 |
| 37% Occipital_Mid_R [52] / 20% Occipital_Sup_R [50] | 0.75776 |
| 78% Lingual_R [48] | 0.97204 |
| 39% Calcarine_R [44] / 27% Cuneus_R [46] | 0.95529 |
| 76% Calcarine_R [44] | 0.92708 |
| 37% Temporal_Sup_L [81] / 35% Rolandic_Oper_L [17] | 0.72006 |
| 93% Occipital_Mid_L [51] | 0.88113 |
| 40% Fusiform_L [55] / 31% Lingual_L [47] | 0.79654 |
| 47% Occipital_Sup_L [49] / 32% Cuneus_L [45] | 0.8983 |
| 49% Occipital_Mid_L [51] / 44% Occipital_Inf_L [53] | 0.74688 |
| 88% Lingual_L [47] | 1 |
| 34% Calcarine_L [43] / 26% Occipital_Mid_L [51] | 0.81339 |
| 38% Lingual_L [47] / 20% Calcarine_L [43] | 0.84961 |
| 67% Calcarine_L [43] | 0.95617 |
| **Brain Region Involved for Salience** | **Intensity** |
| 41% Frontal_Mid_Orb_R [10] / 29% Frontal_Mid_R [8] | 0.74767 |
| 38% Frontal_Inf_Orb_R [16] / 26% Frontal_Mid_Orb_R [10] | 0.76732 |
| 61% Frontal_Mid_R [8] | 0.80919 |
| 84% Frontal_Mid_R [8] | 1 |
| 56% Cingulum_Mid_R [34] / 26% Cingulum_Ant_R [32] | 0.89333 |
| 67% Frontal_Inf_Tri_R [14] | 0.83262 |
| 57% Insula_R [30] / 21% Frontal_Inf_Tri_R [14] | 0.74549 |
| 74% Postcentral_R [58] | 0.73133 |
| 60% Supp_Motor_Area_R [20] / 22% Cingulum_Mid_R [34] | 0.74191 |
| 58% Temporal_Mid_R [86] / 32% Temporal_Sup_R [82] | 0.74898 |
| 50% Temporal_Sup_R [82] / 28% Rolandic_Oper_R [18] | 0.71296 |
| 52% Temporal_Sup_R [82] / 48% Temporal_Mid_R [86] | 0.84572 |
| 66% Temporal_Mid_R [86] | 0.82313 |
| 41% Frontal_Mid_L [7] / 28% Frontal_Sup_L [3] | 0.77442 |
| 75% Frontal_Mid_L [7] | 0.8282 |
| 63% Frontal_Inf_Tri_L [13] | 0.82589 |
| 55% Supp_Motor_Area_L [19] / 24% Frontal_Sup_Medial_L [23] | 0.81985 |
| 61% Frontal_Inf_Tri_L [13] | 0.88701 |
| 77% Temporal_Mid_L [85] | 0.80309 |
| 62% Temporal_Mid_L [85] | 0.81927 |
| 72% Temporal_Mid_L [85] | 0.81848 |
| **Brain Region Involved for Posterior DMN** | **Intensity** |
| 57% Supp_Motor_Area_R [20] / 39% Paracentral_Lobule_R [70] | 0.80048 |
| 63% Postcentral_R [58] | 0.77675 |
| 37% Parietal_Sup_R [60] / 23% Parietal_Inf_R [62] | 0.70656 |
| 80% Precuneus_R [68] | 0.86118 |
| 79% Precuneus_R [68] | 0.74169 |
| 46% Precuneus_R [68] / 37% Cingulum_Mid_R [34] | 0.86742 |
| 51% Postcentral_L [57] / 39% Precentral_L [1] | 0.73914 |
| 61% Postcentral_L [57] | 0.76176 |
| 58% Paracentral_Lobule_L [69] / 35% Precuneus_L [67] | 0.84561 |
| 66% Parietal_Sup_L [59] | 0.92041 |
| 75% Precuneus_L [67] | 1 |
| 49% Parietal_Inf_L [61] / 38% Postcentral_L [57] | 0.7002 |
| 66% Cingulum_Mid_L [33] | 0.70015 |
| 74% Precuneus_L [67] | 0.70861 |
| 46% Precuneus_L [67] / 45% Cingulum_Mid_L [33] | 0.90781 |
| **Brain Region Involved for FPN** | **Intensity** |
| 41% Frontal_Mid_Orb_R [10] / 29% Frontal_Mid_R [8] | 0.71857 |
| 61% Frontal_Mid_R [8] | 0.83527 |
| 84% Frontal_Mid_R [8] | 0.81022 |
| 85% Frontal_Mid_R [8] | 0.89663 |
| 67% Frontal_Inf_Tri_R [14] | 0.72855 |
| 44% Frontal_Inf_Oper_R [12] / 34% Frontal_Inf_Tri_R [14] | 0.76975 |
| 37% Parietal_Sup_R [60] / 23% Parietal_Inf_R [62] | 0.80984 |
| 46% SupraMarginal_R [64] / 34% Parietal_Inf_R [62] | 1 |
| 87% Angular_R [66] | 0.90587 |
| 41% Frontal_Mid_L [7] / 28% Frontal_Sup_L [3] | 0.71232 |
| 40% Frontal_Mid_Orb_L [9] / 39% Frontal_Inf_Orb_L [15] | 0.92596 |
| 63% Frontal_Inf_Tri_L [13] | 0.89738 |
| 44% Frontal_Sup_Medial_L [23] / 43% Frontal_Sup_L [3] | 0.76021 |
| 84% Frontal_Mid_L [7] | 0.96682 |
| 64% Frontal_Inf_Orb_L [15] | 0.88846 |
| 61% Frontal_Inf_Tri_L [13] | 0.83907 |
| 52% Frontal_Inf_Tri_L [13] / 42% Frontal_Inf_Oper_L [11] | 0.9035 |
| 50% Precentral_L [1] / 30% Frontal_Inf_Oper_L [11] | 0.86084 |
| 46% Frontal_Mid_L [7] / 45% Frontal_Sup_L [3] | 0.70241 |
| 81% Precentral_L [1] | 0.70613 |
| 59% Angular_L [65] / 26% Parietal_Inf_L [61] | 0.72952 |
| **Brain Region Involved for subcortical fronto-temporoparietal (Sub-FPTN)** | **Intensity** |
| 38% Frontal_Inf_Orb_R [16] / 26% Frontal_Mid_Orb_R [10] | 0.73524 |
| 45% Frontal_Inf_Tri_R [14] / 28% Frontal_Inf_Orb_R [16] | 0.73048 |
| 67% Frontal_Inf_Tri_R [14] | 0.89288 |
| 44% Frontal_Inf_Oper_R [12] / 34% Frontal_Inf_Tri_R [14] | 0.79496 |
| 37% Parietal_Sup_R [60] / 23% Parietal_Inf_R [62] | 0.7 |
| 46% SupraMarginal_R [64] / 34% Parietal_Inf_R [62] | 0.83401 |
| 58% Temporal_Mid_R [86] / 32% Temporal_Sup_R [82] | 0.75678 |
| 52% Temporal_Sup_R [82] / 48% Temporal_Mid_R [86] | 0.80884 |
| 64% Temporal_Mid_R [86] | 0.87249 |
| 51% Temporal_Inf_R [90] / 47% Temporal_Mid_R [86] | 0.81118 |
| 70% Temporal_Inf_R [90] | 0.90559 |
| 81% Cingulum_Ant_R [32] | 0.72996 |
| 81% Cingulum_Mid_R [34] | 0.79348 |
| 19% Putamen_R [74] / 14% Caudate_R [72] | 0.74234 |
| 52% Thalamus_R [78] | 0.70057 |
| 33% Cingulum_Ant_L [31] / 23% Rectus_MedOFC_L [27] | 0.80333 |
| 40% Frontal_Mid_Orb_L [9] / 39% Frontal_Inf_Orb_L [15] | 0.89789 |
| 63% Frontal_Inf_Tri_L [13] | 0.89456 |
| 84% Frontal_Mid_L [7] | 0.79996 |
| 64% Frontal_Inf_Orb_L [15] | 0.82283 |
| 61% Frontal_Inf_Tri_L [13] | 0.76427 |
| 52% Frontal_Inf_Tri_L [13] / 42% Frontal_Inf_Oper_L [11] | 0.82259 |
| 50% Precentral_L [1] / 30% Frontal_Inf_Oper_L [11] | 0.77428 |
| 52% Cingulum_Mid_L [33] / 45% Supp_Motor_Area_L [19] | 0.72432 |
| 59% Angular_L [65] / 26% Parietal_Inf_L [61] | 0.77823 |
| 55% Temporal_Mid_L [85] / 27% Angular_L [65] | 0.76983 |
| 57% Parietal_Inf_L [61] / 36% SupraMarginal_L [63] | 0.84927 |
| 82% Temporal_Mid_L [85] | 1 |
| 63% Temporal_Inf_L [89] | 0.97964 |
| 37% Cingulum_Mid_L [33] / 36% Cingulum_Ant_L [31] | 0.86863 |
| 53% Thalamus_L [77] / 0% Thalamus_R [78] | 0.70005 |
